# Treatment of giant cervico-mediastinal lymphatic malformations: a case series

**DOI:** 10.1186/s13256-018-1705-0

**Published:** 2018-06-15

**Authors:** So-Hyun Nam, Kyoung-Ah Kwon

**Affiliations:** 1 Division of Pediatric Surgery, Department of Surgery, Dong-A University College of Medicine, Dong-A Medical Center, 26 Daesingongwon-Ro, Seo-Gu, Busan, 49201 South Korea; 2 Department of Pediatrics, Dong-A University College of Medicine, Dong-A Medical Center, Busan, South Korea

**Keywords:** Lymphatic malformations, Neck, Mediastinum

## Abstract

**Background:**

Lymphatic malformations are histologically benign vascular structures that vary in anatomic lesion and size. Extensive head and neck lymphatic malformations may be life-threatening. In the present study, we described three difficult-to-treat infants with giant cervico-mediastinal lymphatic malformations accompanied by severe respiratory distress.

**Case presentations:**

Case 1. A Korean girl born at a gestational age of 37 weeks and weighing 2920 g had a large cervical mass compressing the trachea. Despite initial OK-432 sclerotherapy, the mass extended over the contralateral retropharyngeal space and mediastinum. Although the cervical mass was completely excised, our patient was not weaned off the ventilator. The mediastinal lymphatic malformation was excised, and our patient underwent continued intensive respiratory care with nasal continuous positive airway pressure for 6 months. She is now 5 years old and doing well without any sequelae. Case 2. A 5-month-old Korean boy showed respiratory difficulty with feeding intolerance after partial excision of a neck lymphatic malformation. We found that the remnant cervical mass had grown into the retropharyngeal space and mediastinum. After a second operation for the cervico-mediastinal mass, he experienced severe respiratory difficulty requiring ventilator care for 6 months. However, he died from central-line fungal sepsis. Case 3. A 30-day-old Korean girl was referred for remnant lymphatic malformation after partial excision. The cervical mass extended to the mediastinum and occupied half of the thoracic cavity, encasing all of the major vessels. After surgical excision, she underwent ventilator care and pleurodesis three times with doxycycline for recurrent pleural effusion. At the age of 26 months, she was weaned off supplementary oxygen and she showed normal development without any sequelae.

**Conclusions:**

Despite difficulties in the treatment process, combinations of delicate surgical treatment, appropriate adjuvant sclerotherapy, and intensive respiratory supportive care could result in a good outcome. However, complications due to long-term intensive care could still be considered.

## Background

Lymphatic malformations (LMs) are benign vascular lesions that arise from disturbances during embryologic development of the lymphatic system. It can form variable-sized cystic lesions within lymphatic channels [1–5]. Most of these lesions are in the head and neck area (80–90%), but they can appear anywhere in the body, including in the extremities, trunk, abdomen, retroperitoneum, and thorax [1, 3–5]. Although histologically benign, these lesions are regarded as clinically intractable because disease progression would not be expectable and treatment could be supportive rather than curative. Small-sized LMs may be asymptomatic even if identified during radiologic examination. Symptoms can be serious if the malformations are large, compress surrounding structures, or invade vital organs. In particular, head and neck LMs involving the oral cavity, pharynx, larynx, esophagus, and/or trachea may be life-threatening. The treatment of choice, such as observation, excision, or sclerotherapy, depends on many factors, including associated symptoms and complications, disfigurement, and the surgeon’s experience [1–5]. Surgery is the definitive treatment, but complete excision is often not feasible because of the anatomic location of these lesions and their tendency to infiltrate surrounding tissue [3, 6]. In addition, LMs may widely displace or entirely envelop nerves and blood vessels; they may also cross fascial boundaries and distort normal anatomy [6–8]. This report describes three patients with giant cervico-mediastinal LMs accompanied by severe respiratory distress. We discuss the difficulties and the lessons learned during the treatment process.

## Case presentations

**Fig. 1:**
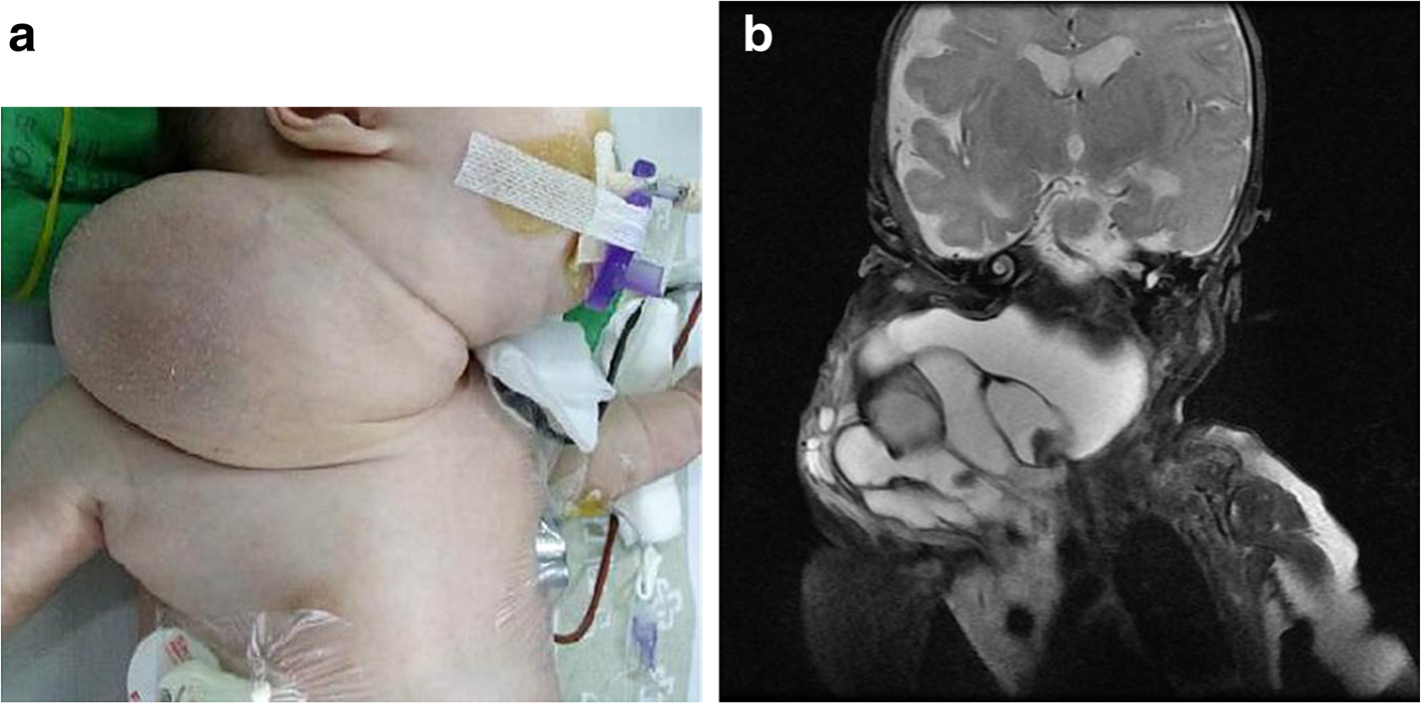
**a** Case 1. Photograph of a 41-day-old infant with a large cervical lymphatic malformation. **b** Case 1. Large cervico-mediastinal lymphatic malformations that encased almost all of the cervical vital structures, causing airway obstruction, visualized on magnetic resonance imaging

**Fig. 2:**
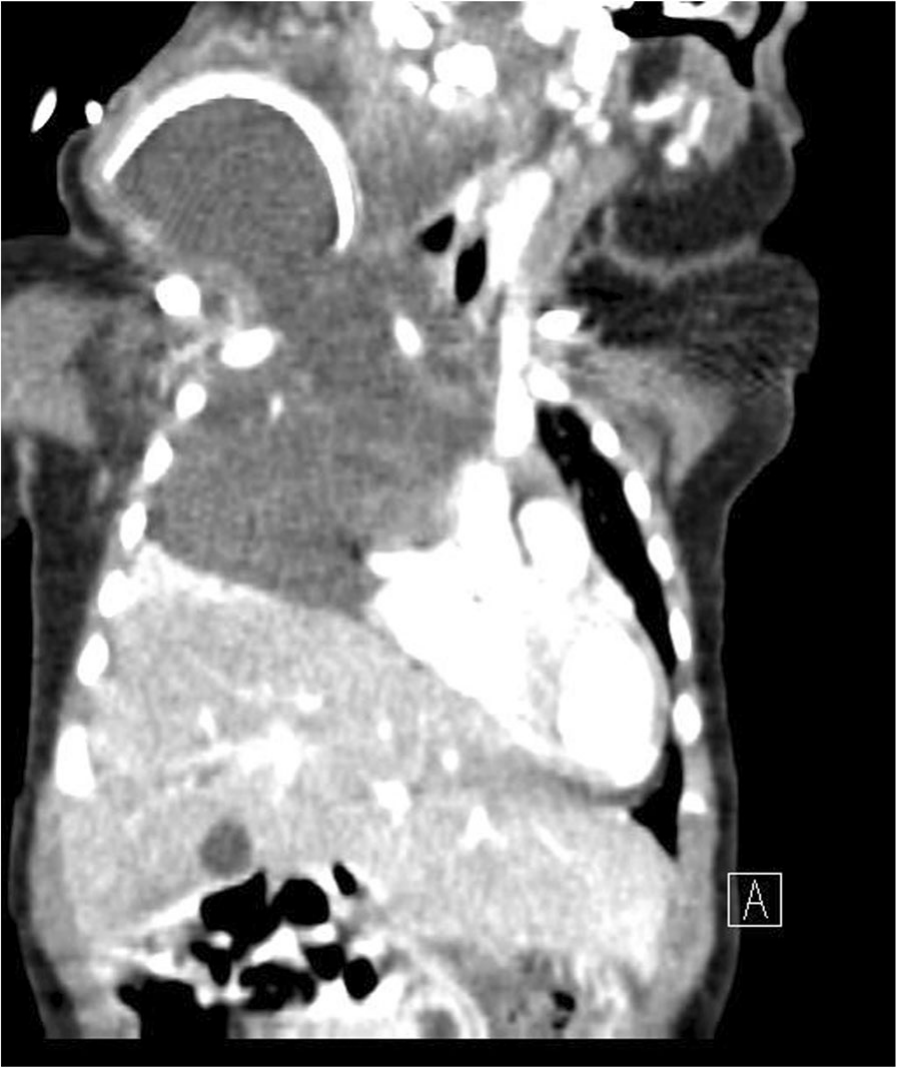
Case 1. Enlarged mediastinal mass after cervical lymphatic malformation excision, visualized on a computed tomography scan

Patient 1. A Korean girl was born at a gestational age of 37 weeks and weighed 2920 g. She was prenatally diagnosed with a cervical mass on the right side of her neck (Fig. 1a). Magnetic resonance imaging (MRI) showed a multi-septated cystic mass (5 × 8 × 7 cm) that encased almost all of the cervical vital structures. With a diagnosis of LM, she was treated 14 days after birth with OK-432 at another institution. Despite this treatment, the mass continued to compress the trachea and extended over the contralateral retropharyngeal space and mediastinum (Fig. 1b). The airway could not be maintained without an endotracheal tube. She was transferred to our institution at the age of 23 days. Because of the huge neck mass on her right side, her face was tilted to the left. There was no gross deformity or neurologic impairment. Laboratory findings revealed a hemoglobin level of 8.2 g/dL, white blood cell count of 24.1 × 10^3^/mm^3^, and platelet count of 938 × 10^3^/mm^3^. Blood chemistry profile and electrolyte level results showed normal ranges, but her C-reactive protein was elevated as 9.17 mg/dL. At the age of 26 days, we excised the cervical lesion completely without injury to the vital organs, major vessels, and nerves. We dissected the mass along the thin wall of the LM and preserved the nerves and vessels as much as possible. The remaining tissues were ligated with silk ties to minimize lymphatic leakage. Histologically, LM was verified. However, our patient could not be weaned off a ventilator, and the mediastinal LM became larger. Our patient was treated twice with sclerotherapy with doxycycline, but it was ineffective (Fig. 2). At the age of 4 months, she underwent mediastinal LM excision via a right thoracotomy. We were able to remove the mass completely while preserving the vessels, accompanied by concurrent diaphragm plication because of the phrenic nerve injury. Postoperatively, the right lung became atelectatic with a large amount of pleural effusion. We continued extensive physiotherapy and repeatedly drained the pleural effusion percutaneously. Intensive respiratory care with nasal continuous positive airway pressure (CPAP) for 6 months allowed our patient to be weaned off assisted ventilation. She was discharged without supplemental oxygen at the age of 11 months. She is now 5 years old and doing well without any sequelae.

**Fig. 3:**
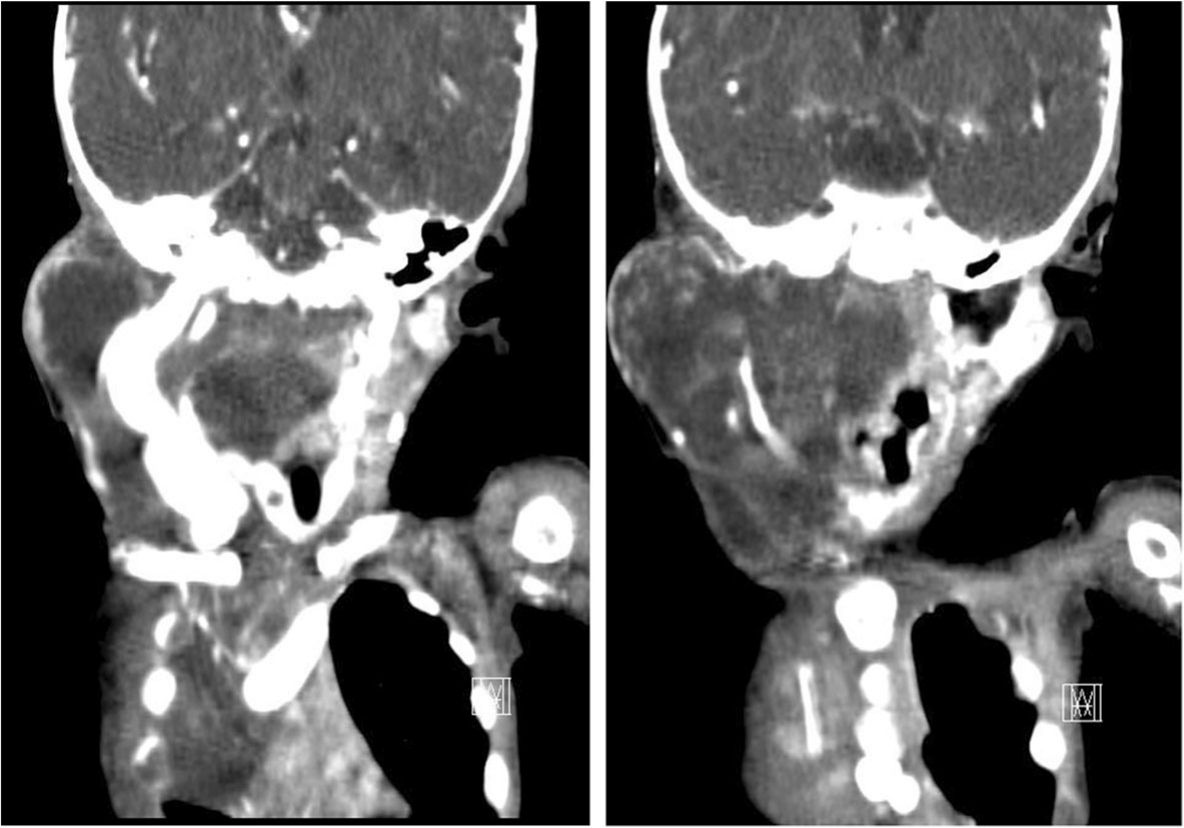
Case 2. Cervico-mediastinal lymphatic malformations after partial excision, visualized on a computed tomography scan

Patient 2. A Korean boy born at a gestational age of 37 weeks weighing 3200 g underwent partial excision of a neck LM at another hospital. However, the remnant cervical mass regrew rapidly and caused feeding intolerance and respiratory difficulty. On initial physical examination at 4 months old, he looked very sick and his body weight was 4.2 kg. His respiration was rapid, shallow, and the breathing sound was coarse. His tongue was severely dehydrated. The right neck mass was very hard and the overlying skin showed wound contracture. He did not show neurological impairment. An initial blood gas analysis showed that pCO_2_ was 48 mmHg and pO_2_ was 92 mmHg. His hemoglobin level was 8.9 g/dL white blood cell count was 9.8 × 10^3^/mm^3^, and platelet count was 350 × 10^3^/mm^3^. His serum total protein level was 3.6 g/Land his albumin level was 2.1 g/dL. Other blood chemistry profile and electrolyte level results showed normal ranges. His airway was deviated by a cervical LM with internal hemorrhage, and the mediastinal LM became enlarged, occupying approximately half of the right thoracic cavity, as seen on the computed tomography scan (Fig. 3). At the age of 5 months, the cervical LM was mostly removed. It was very difficult because the previous excision scar and adjacent tissue were too firm to dissect. The retropharyngeal LM remained because it was very deep and unreachable. We delayed mediastinal LM excision because the cervical lesion operation took over 6 h. Two weeks later, the right mediastinal LM was removed. The mass encasing the superior vena cava and subclavian vein also compressed the trachea and esophagus. After the second operation, our patient experienced severe respiratory difficulty requiring ventilator care for 6 months. The upper lobe of his right lung had almost completely collapsed without fluid collection. After 8 months in the intensive care unit (ICU), our patient was transferred to the general ward while receiving nasal CPAP. However, he died from central-line fungal sepsis while being prepared for discharge from the hospital at the age of 14 months.

**Fig. 4:**
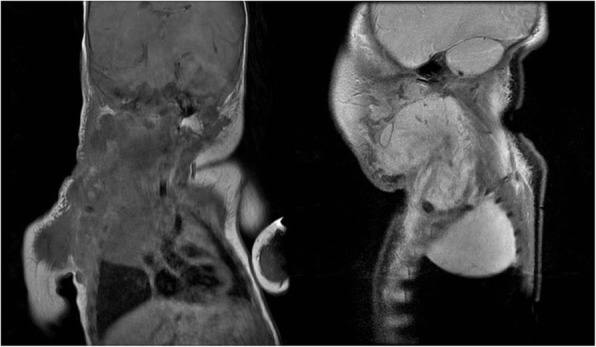
Case 3. Cervico-mediastinal lymphatic malformations after partial excision, visualized on magnetic resonance imaging (coronal and sagittal images)

Patient 3. A Korean girl born at a gestational age of 40 weeks weighing 3460 g had been prenatally diagnosed with a right neck cervico-mediastinal mass. MRI and ultrasonography revealed multi-septated cystic LMs in the submandibular, carotid, posterior cervical spaces of the right neck, both prevertebral and retropharyngeal spaces, right lower anterior neck, and mediastinum. Seven days after birth, the neck mass was partially excised at another hospital. However, the remaining mass grew rapidly and was accompanied by submental swelling. The cervical mass extended to the mediastinum, occupied half of the thoracic cavity, and encased all of the major vessels (Fig. 4). At the age of 29 days, she looked grossly normal without neurologic impairment and she cried well. Her body weight was 4.0 kg. The right neck mass was soft in the submandibular area, but the posterolateral lesion was very hard. Initial blood gas analysis showed that pCO_2_ was 31.5 mmHg and pO_2_ was 96.1 mmHg. Her hemoglobin level was 10.0 g/dL white blood cell count was 7.8 × 103/mm^3^, and platelet count was 140 × 103/mm^3^. Blood chemistry profile and electrolyte level results were within normal ranges. At the age of 30 days, we removed the cervical and mediastinal LMs simultaneously. The lesion occupying the parotid gland was left untouched to avoid facial nerve injury. Despite preservation of the phrenic nerve, she experienced diaphragm palsy with a collapsed right lung. She underwent diaphragmatic plication 1 month later but continued to experience pneumothorax and recurrent pleural effusion. She underwent pleurodesis three times with doxycycline accompanied by oxygen support with a high-flow nasal cannula. She was discharged at the age of 4 months with supplementary oxygen at 1 L/min via nasal prongs. At the age of 9 months, we observed enlargement of the remnant cervical LM, which formed a macrocystic lesion. We performed sclerotherapy with doxycycline. At the age of 26 months, she was weaned off supplementary oxygen and she showed normal development without any sequelae.

## Discussion

We described three cases of cervico-mediastinal lymphatic malformations requiring extensive surgery with long-time respiratory supportive treatment. Previous reports in the literature were sporadic and varied in age. This report only includes neonates with respiratory compromise caused from cervico-mediastinal LM. We wanted to share a difficult treatment course.

Two types of treatment are available for LMs: sclerotherapy and surgery. Surgical intervention is the best method for complete resection, but it is often difficult and insufficient. Surgery can damage the surrounding structures, particularly the nerves and blood vessels, and can cause scarring; sometimes, there is recurrence due to incomplete excision [2, 9–11]. Complete excision of LM with vital organs, such as the tongue, pharynx, and orbit, should not be performed. Thus, intralesional sclerotherapy using bleomycin, tetracycline, alcohol, and OK-432 should be the treatment of choice [2, 12]. Sclerotherapy can induce endothelial inflammation in vascular structures, resulting in thrombosis, occlusion, and fibrosis [1, 2, 13]. OK-432, which is derived from a strain of *Streptococcus pyogenes*, is usually the first choice for a sclerosing agent [14]. Although effective for single cystic lesions and macrocystic lesions, OK-432 has been found to be less effective for microcystic and cavernous lesions [2]. OK-432, however, may be useful as neoadjuvant treatment before surgical excision and as postoperative adjuvant treatment following incomplete excision. In addition, it reduces lymphatic fluid retention after surgery [3]. We thought that sclerotherapy and surgical treatment could complement each other.

None of these patients could be treated by sclerotherapy alone because all the masses were large and deep with areas that were inaccessible to intralesional injection of sclerosing agents. The first line of treatment, comprising partial excision or OK-432 sclerotherapy, was ineffective and exacerbated respiratory symptoms. We made a decision that extensive excision for cervical lesions was necessary for airway management. It was successful, but we confronted persistent pulmonary dysfunction. Unlike the previous reports of asymptomatic mediastinal LMs connected to cervical lesions, these lesions caused severe respiratory distress with a mass effect in the thoracic cavity. We found that the mediastinal LMs in this series were mostly composed of microcystic lesions. The clear and tiny vesicles made it denser and harder than macrocystic lesions [1]. Moreover, these malformations infiltrate more and are difficult to resect surgically [8, 14, 15]. While the LMs in the neck were relatively soft and cystic, the LMs in the mediastinum were very hard, without the cystic portion. It definitely seemed to interfere with respiratory function. We learned that mediastinal LMs extending from cervical LMs can affect respiratory function, and they required surgical intervention.

Partial resection of a large LM was not beneficial to our patients. It resulted in severe fibrosis and adhesion of the overlying skin and subcutaneous tissue. The remaining LM did not grow outward but grew into the head and neck cavity, severely compressing the airway and esophagus. The overall recurrence rate after surgical treatment ranges from 35 to 64%, with at least 17% of LMs recurring even after complete removal [16, 17]. Postoperatively, remnant lymphatic channels dilate with marked regenerative ability [3, 18]. Lesion regeneration has been associated with increased activity of vascular endothelial growth factor and decreased activity of pigment epithelium-derived factor [19]. Thus, partial resection was not ideal for our cases.

Of note, it took considerable time for patients to recover pulmonary function after mediastinal mass removal. The reasons for this delay are unclear, although surgical removal of a mediastinal LM relieves compression on the lung parenchyma. One possible reason is the presence of reactive fluid from the cut surface of the lymphatics and postoperative swelling, which could generate another space-occupying lesion in the thoracic cavity. Another possibility is that the mediastinal mass in the thoracic cavity interferes with the development of normal lung parenchyma during the fetal period, which could affect overall pulmonary function. Fortunately, we found that positive pressure support sufficiently improves gas exchange. Although patients were on assisted ventilation for a long time, they could be weaned off supplementary oxygen.

This series describes three difficult-to-treat patients with extensive cervico-mediastinal LMs. The two surviving patients will require careful long-term follow-up for recurrence and pulmonary function. Despite difficulties during the treatment process, combinations of delicate surgical treatment, appropriate adjuvant sclerotherapy, and intensive respiratory supportive care resulted in a good outcome. However, complications due to long-term intensive care could still be considered.

## Conclusions

Giant cervico-mediastinal lymphatic malformations in infants could be treated with surgical removal and adjuvant sclerotherapy. Overall, pulmonary dysfunction caused from mediastinal lymphatic malformations could be overcome with long-time respiratory supportive care.

## References

[1] Elluru RG, Balakrishnan K, Padua HM. Lymphatic malformations: diagnosis and management. Semin Pediatr Surg. 2014;23:178–85. 10.1053/j.sempedsurg.2014.07.002 25241095

[2] Okazaki T, Iwatani S, Yanai T, *et al*. Treatment of lymphangioma in children: our experience of 128 cases. J Pediatr Surg. 2007;42:386–9. 10.1016/j.jpedsurg.2006.10.012 17270554

[3] Kim SY, Lee S, Seo JM, *et al*. Postoperative adjuvant OK-432 sclerotherapy for treatment of cervicofacial lymphatic malformations: an outcomes comparison. Int J Pediatr Otorhinolaryngol. 2015;79:570–5. 10.1016/j.ijporl.2015.01.030 25698461

[4] Hochman M, Adams DM, Reeves TD. Current knowledge and management of vascular anomalies, II: malformations. Arch Facial Plast Surg. 2011;13:425–33. 10.1001/archfacial.2011.795 22106189

[5] Elluru RG, Azizkhan RG. Cervicofacial vascular anomalies. II. Vascular malformations. Semin Pediatr Surg. 2006;15:133–9. 10.1053/j.sempedsurg.2006.02.011 16616317

[6] Lee GS, Perkins JA, Oliaei S, *et al*. Facial nerve anatomy, dissection and preservation in lymphatic malformation management. Int J Pediatr Otorhinolaryngol. 2008;72:759–66. 10.1016/j.ijporl.2008.01.034 18378008

[7] Bhatt N, Perakis H, Watts TL, *et al*. Traumatic hemorrhage and rapid expansion of a cervical lymphatic malformation. Ear Nose Throat J. 2011;90:20–2. 10.1177/014556131109000106 21229505

[8] Chiara J, Kinney G, Slimp J, *et al*. Facial nerve mapping and monitoring in lymphatic malformation surgery. Int J Pediatr Otorhinolaryngol. 2009;73:1348–52. 10.1016/j.ijporl.2009.06.008 19592118

[9] Emery PJ, Bailey CM, Evans JN. Cystic hygroma of the head and neck: a review of 37 cases. J Laryngol Otol. 1984;98:613–9. 10.1017/s0022215100147176 6736759

[10] Riechelmann H, Muehlfay G, Keck T, *et al*. Total, subtotal, and partial surgical removal of cervicofacial lymphangiomas. Arch Otolaryngol Head Neck Surg. 1999;125:643–8. 10.1001/archotol.125.6.643 10367920

[11] Kennedy TL, Whitaker M, Pellitteri P, *et al*. Cystic hygroma/lymphangioma: a rational approach to management. Laryngoscope. 2001;111:1929–37. 10.1097/00005537-200111000-00011 11801972

[12] Brewis C, Pracy JP, Albert DM. Treatment of lymphangiomas of the head and neck in children by intralesional injection of OK-432 (Picibanil). Clin Otolaryngol Allied Sci. 2000;25:130–4. 10.1046/j.1365-2273.2000.00338.x 10816217

[13] Ogita S, Tsuto T, Nakamura K, *et al*. OK-432 therapy in 64 patients with lymphangioma. J Pediatr Surg. 1994;29:784–5. 10.1016/0022-3468(94)90370-0 8078021

[14] Raveh E, deJong AL, Taylor GP, *et al*. Prognostic factors in the treatment of lymphatic malformations. Arch Otolaryngol Head Neck Surg. 1997;123:1061–5. 10.1001/archotol.1997.01900100035004 9339981

[15] Tran Ngoc N, Tran Xuan N. Cystic hygroma in children: a report of 126 cases. J Pediatr Surg. 1974;9:191–5. 10.1016/s0022-3468(74)80120-x 4825790

[16] Greene AK, Perlyn CA, Alomari AI. Management of lymphatic malformations. Clin Plast Surg. 2011;38:75–82. 10.1016/j.cps.2010.08.006 21095473

[17] Fliegelman LJ, Friedland D, Brandwein M, *et al*. Lymphatic malformation: predictive factors for recurrence. Otolaryngol Head Neck Surg. 2000;123:706–10. 10.1067/mhn.2000.110963 11112962

[18] Lei ZM, Huang XX, Sun ZJ, *et al*. Surgery of lymphatic malformations in oral and cervicofacial regions in children. Oral Surg Oral Med Oral Pathol Oral Radiol Endod. 2007;104:338–44. 10.1016/j.tripleo.2006.12.025 17428699

[19] Sidle DM, Maddalozzo J, Meier JD, *et al*. Altered pigment epithelium-derived factor and vascular endothelial growth factor levels in lymphangioma pathogenesis and clinical recurrence. Arch Otolaryngol Head Neck Surg. 2005;131:990–5. 10.1001/archotol.131.11.990 16301371

